# Identification and characterization of microRNAs in the ovaries of multiple and uniparous goats (Capra hircus) during follicular phase

**DOI:** 10.1186/1471-2164-15-339

**Published:** 2014-05-06

**Authors:** Ying-Hui Ling, Chun-Huan Ren, Xiao-Fei Guo, Li-Na Xu, Ya-Feng Huang, Jian-Chuan Luo, Yun-Hai Zhang, Xiao-Rong Zhang, Zi-Jun Zhang

**Affiliations:** College of Animal Science and Technology, Anhui Agricultural University, No. 130 Changjiang west road, Hefei, 230036 P.R. China; Anhui Provincial Laboratory of Local Animal Genetic Resources Conservation and Biobreeding, No. 130 Changjiang west road, Hefei, 230036 P.R. China; Institute of Plant Protection and Agro-Products Safety, Anhui Academy of Agricultural Sciences, No. 40 South Nongke Road, Hefei, 230031 P.R. China

**Keywords:** MicroRNA, Kidding rate, Solexa sequencing, Ovary, Follicular phase, Goat

## Abstract

**Background:**

Superior kidding rate is an important economic trait in production of meat goat, and ovulation rate is the precondition of kidding rate. MicroRNAs (miRNAs) play critical roles in almost all ovarian biological processes, including folliculogenesis, follicle development, follicle atresia, luteal development and regression. To find out the different ovarian activity and follicle recruitment with miRNA-mediated posttranscriptional regulation, the small RNAs expressed pattern in the ovarian tissues of multiple and uniparous Anhui White goats during follicular phase was analyzed using Solexa sequencing data.

**Results:**

1008 miRNAs co-expressed, 309 and 433 miRNAs specifically expressed in the ovaries of multiple and uniparous goats during follicular phase were identified. The 10 most highly expressed miRNAs in the multiple library were also the highest expressed in the uniparous library, and there were no significantly different between each other. The highest specific expressed miRNA in the multiple library was miR-29c, and the one in the uniparous library was miR-6406. 35 novel miRNAs were predicted in total. GO annotation and KEGG Pathway analyses were implemented on target genes of all miRNA in two libraries. RT-PCR was applied to detect the expression level of 5 randomly selected miRNAs in multiple and uniparous hircine ovaries, and the results were consistent with the Solexa sequencing data.

**Conclusions:**

In the present study, the different expression of miRNAs in the ovaries of multiple and uniparous goats during follicular phase were characterized and investigated using deep sequencing technology. The result will help to further understand the role of miRNAs in kidding rate regulation and also may help to identify miRNAs which could be potentially used to increase hircine ovulation rate and kidding rate in the future.

**Electronic supplementary material:**

The online version of this article (doi:10.1186/1471-2164-15-339) contains supplementary material, which is available to authorized users.

## Background

MicroRNAs (miRNAs) are a group of endogenous ~22 nt small non-coding RNAs that can modulate gene expression by inhibiting mRNA translation or regulating mRNA degradation at the post-transcriptional level [1]. It was once estimated that known miRNAs account for around 1% of predicted genes in higher eukaryotic genomes and that up to 30% of genes might be regulated by miRNAs [2]. MiRNAs regulation have been implicated in varied physiological processes including cell proliferation [3], differentiation [4], apoptosis [5], tumorigenesis [6], hormone secretion [7], metabolism [8] and reproduction control [9]. On goat’s miRNA study, researchers have focused their interest on mammary gland of dairy goat [10], hair follicle of cashmere goat [11] and muscle or reproduction of meat goat [12, 13]. A recent study showed that over-expression of miR-103 in goat’s mammary gland epithelial cells increased transcription of genes associated with milk fat synthesis, resulted in an up-regulation of fat droplet formation, triglyceride accumulation, and the proportion of unsaturated fatty acids [14]. MiRNAs research in animal ovary has been extensively explored with the improvement of study methods. MiRs-31 and MiRs-92 were discovered in pig ovary by homology analysis and confirmed with northern blot [15]. Cloning technique had been adopted to identify miRNAs expressed in mice ovary, obtained a total of 122 miRNAs from the ovaries of 2-wk-old and adult mice [16]. A mouse mutant with a ~75% loss of Dicer1 miRNA levels was predicted to cause the decreasing of angiogenesis in the corpus luteum ultimately resulted in female infertility. On further study, the miR-17-5p and let-7b which regulate the expression of tissue inhibitor of metalloproteinase 1 had been proved contributed to the mutant mouse’s infertility [17]. In 2013, the different expression of miRNAs in the ovaries of pregnant and non-pregnant Anhui White goats was identified and analyzed, 617 conserved and 7 putative novel miRNA in hircine ovaries had been detected by high-throughput sequencing [13].

Superior kidding rate is an important economic trait in production of meat goat, while ovulation rate is the precondition of kidding rate [18]. Follicular phase accompanied by the increasing of FSH, is the phase including of follicle recruitment and dominant follicle development. Multiple follicle development and ovulation have been attributed to higher elevations of FSH above the threshold level [19–21]. Researchers had discovered that bone morphogenetic protein 15 (BMP15) and growth differentiation factor 9 (GDF9) contribute to all stages of follicular development including activation of the primordial follicles [22, 23]. James et al. mutated the genes for oocyte-derived growth factors GDF9 and BMP15 which were associated with both increased ovulation rate and sterility in Cambridge and Belclare sheep [24, 25]. There are also specific genes, for example the FecB or Booroola gene, that result in ovulation rates greater than five [18, 26]. In recent years, many studies indicated that miRNAs play critical roles in almost all ovarian biological processes, including folliculogenesis, follicle development, follicle atresia, luteal development and regression [17, 27–31]. The Anhui white goat is known for its precocious puberty, higher fertility, and higher leather quality compared with other types of goat. Anhui white goat ewes can estrus all year round. The average Anhui white goat kidding rate is 227-239% which is belong to varieties of high kidding rate in goats. It is therefore an ideal model for the study of goat breeding traits.

In the present study, we characterized and investigated the differential expression of miRNAs in the ovaries of multiple and uniparous Anhui White goats using deep sequencing technology. The result will help to further understand the role of miRNAs in kidding rate regulation and also may help to identify miRNAs which could be potentially used to increase hircine ovulation rate and kidding rate in the future.

## Results

### Overview of sequencing data

**Figure 1:**
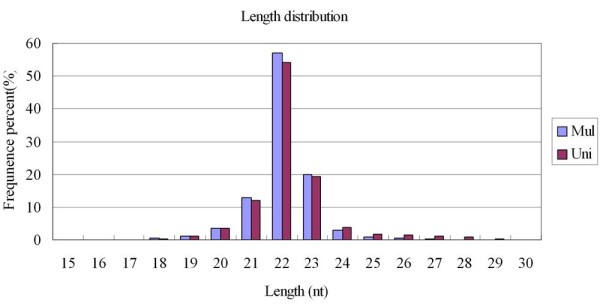
**Frequency distribution of sequence lengths of the sequencing results.**

In order to identify differentially expressed miRNA during follicular phase in the ovaries of multiple and uniparous Anhui White goats, two small RNA libraries were constructed by Solexa sequencing. A total of 12,000,000 raw reads were obtained. After discarding the sequences shorter than 18 nt, eliminating low-quality sequences and removing contaminants formed by adapter-adapter ligation, reads without 3’ligation and insert tags were obtained. Ultimately, 5,948,837 and 5,945,145 clean reads which obtained from multiple and uniparous goats remained for further analysis (Table 1). Subsequently, all identical sequence reads were classified as groups, and 160,284 and 235,735 unique sequences were obtained (Additional file 1). The length distribution of the reads was similar between the two libraries (Figure 1). The majority of the small RNA were 20-24 nt range. Sequences 22 nt in length, the typical size of Dicer-derived products [32], peaked at length distribution, respectively accounted for 56.93% and 54.26% of the total sequence reads in the multiple and uniparous libraries.

**Table 1:** **The classification of total small RNA tags by Solexa sequencing**

Type	Mul		Uni		Total
	Counts	Percent	Counts	Percent	
Total_reads	6,000,000		6,000,000		12,000,000
High_quality	5,965,487	100%	5,963,795	100%	11,929,282
3'adapter_null	973	0.02%	1,066	0.02%	2,039
Insert_null	354	0.01%	665	0.01%	1,019
5'adapter_contaminants	9,183	0.15%	10,495	0.18%	19,678
Smaller_than_18nt	6,119	0.10%	6,375	0.11%	12,494
PolyA	21	0.00%	49	0.00%	70
Clean_reads	5,948,837	99.72%	5,945,145	99.69%	11,893,982

**Figure 2:**
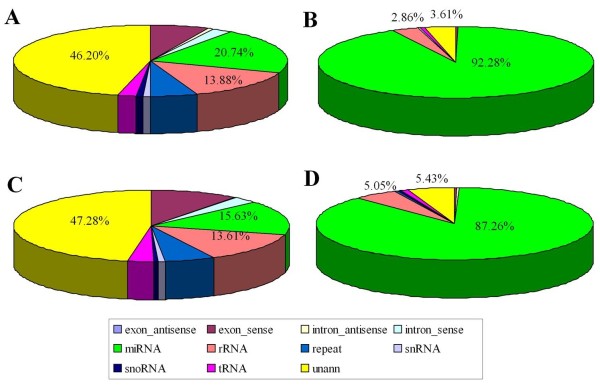
**Composition of small RNA classes of the Solexa.** Note: **(A)** Total number of unique sequences in the multiple library. **(B)** Total number of reads in the multiple library. **(C)** Total number of unique sequences in the uniparous library. **(D)** Total number of reads in the uniparous library.

For assessing the efficiency of Solexa sequencing and the quality of sequence itself, all of the clean reads were annotated and classified by aligning against the Rfam10.1 database, Genbank and the miRBase20.0 database. However, some sRNA tags may be mapped to more than one category. To make every unique small RNAs mapped to only one annotation, we followed the following priority rule: rRNAetc (Genbank > Rfam) > known miRNA > repeat > exon > intron [33]. All of the clean reads were divided into the following categories: exon_antisense, exon_sense, intron_antisense, intron_sense, miRNA, rRNA, repeat, scRNA, snRNA, snoRNA, srpRNA, tRNA, unan (sequences were not mapped to any known reference databases). The composition of the RNA classes in each library was shown in Figure 2 and Additional file 1. The proportion of total rRNA is a mark for sample quality check. Usually it should be less than 60% in plant samples [34] and 40% in animal samples as high quality (unpublished data by BGI). The proportion of total rRNA was 2.86% and 5.05% in multiple and uniparous librariy respectively, indicating that the ovaries samples collected were of high quality in this study. In order to analyze the two libraries expression and distribution, all of the clean Solexa reads were mapped to the goat genome sequence using SOAP software. In the clean reads of multiple and uniparous libraries, 4,780,962 reads (account for 80.37%) and 4,705,068 reads (account for 79.14%) were mapped to the goat genome (Additional file 1). Conserved miRNAs accounted for 92.28% and 87.26% of the total clean reads, and accounted for 20.74% and 15.63% of the unique reads (Figure 2) in the multiple and uniparous small RNA libraries, respectively. The result of two libraries showed that the majority of total reads was classified as miRNA, which suggested that the sequencing of present study was successful. However, the highest fraction of unique reads was attributed to unann, and the length distribution of its small RNA was mainly round about 20-24 nt (Additional file 2). Therefore, just like which had be reported before that there are still many kind of miRNA waiting to be found for us [13].

### Differential expression of conserved miRNAs in the ovaries of multiple and uniparous goats during follicular phase

Since there is no miRNAs information of goat in the miRbase 20.0 database, we aligned the clean reads to the miRNA precursor/mature miRNAs of all known animals in the miRBase 20.0 database. The sequence and count of families (no specific species) were obtained which could be found in the two libraries (Additional file 3). Considering little mismatches between sequences, 1317 and 1441 conserved miRNAs were identified in the multiple and uniparous libraries. Among them, 1008 miRNAs were co-expressed, 309 and 433 miRNAs were specifically expressed in the multiple and uniparous libraries, respectively. The overwhelming majority of these specifically expressed miRNAs’ expression level were very low (under 5), whereas the expression level of 4 (miR-29c, miR-1996b, miR-3135b, miR-3934-5p) and 9 (miR-6406, miR-6317, miR-4001e-3p, miR-1692, miR-6215, miR-4674, miR-1591-3p, miR-4090-3p, miR-1589) specific expressed miRNAs in multiple and uniparous libraries were higher than 1000 (Table 2). The highest specific expressed miRNA in multiple library was miR-29c which reached counts of 5,214 (normalized expression level of 876), and the highest specific expressed miRNA in uniparous library was miR-6406 which reached counts of 42,571 (normalized expression level of 7,161).

**Table 2:** **The expression level of specific expressed miRNAs which were higher than 1000**

	Mul			Uni	
MiRNA	Counts	Mul-std.	MiRNA	Counts	Uni-std.
miR-29c	5214	876	miR-6406	42571	7161
miR-1996b	1771	298	miR-6317	5655	951
miR-3135b	1508	253	miR-4001e-3p	3644	613
miR-3934-5p	1212	204	miR-1692	3028	509
			miR-6215	2394	403
			miR-4674	2030	341
			miR-1591-3p	1991	335
			miR-4090-3p	1800	303
			miR-1589	1395	235

Note: -std. represents normalized expression level of miRNA in a library.

**Figure 3:**
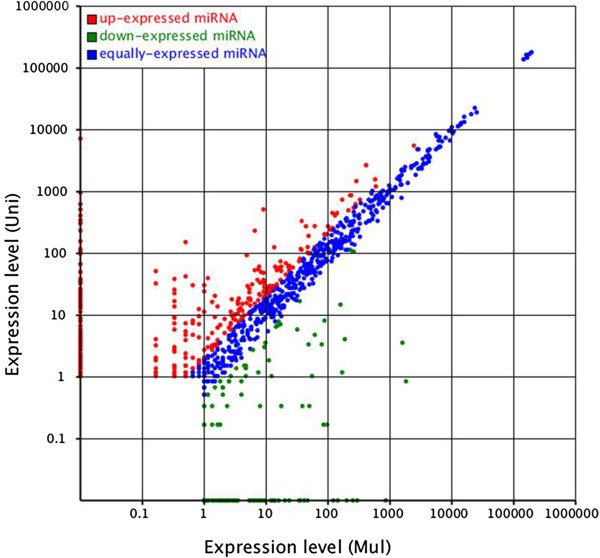
**Differences of miRNA expression between the two libraries.** Note: The scatter plot of differentially expressed miRNAs (control: X-axis, treatment: Y-axis). The X and Y show the expression level of miRNAs in the two samples respectively. Red points represent miRNAs with ratio > 2; Blue points represent miRNAs with 1/2 < ratio ≤ 2; Green points represent miRNAs with ratio ≤ 1/2. Ratio = normalized expression of the treatment/normalized expression of the control.

The differentially expressed miRNAs between the two libraries were showed in Figure 3 and Additional file 4. The same as previous study on miRNA detecting [10, 11, 13, 27, 35, 36], most of its expression quantity were equivalent, while there were also some miRNAs expressed differently between the two experimental group (Figure 3). 60.7% of the miRNAs expression was not significant, 3.6% of the miRNAs were significantly different (0.01 ≤ p < 0.05) and 35.7% of the miRNAs were significantly different (p < 0.01) in the multiple and uniparous libraries. The 10 most highly expressed miRNAs (let-7b, let-7b-5p, let-7-5p, let-7c, let-7c-5p, let-7f-5p, let-7f, let-7, miR-140, miR-320a) in the multiple library were also the highest expressed in the uniparous library, and there were no significantly different between each other.

MiRNAs clustered together for similar expression patterns [37]. In Additional file 5, green indicates that the miRNA has higher expression level in multiple library, red indicates that the miRNA has higher expression in uniparous library. All differentially expressed miRNAs in two libraries clustered together after 6 rounds of clustering.

### Identification of potential novel miRNAs

The characteristic hairpin structure of miRNA precursor can be used to predict novel miRNA. The novel miRNAs were predicted by Mireap software (http://sourceforge.net/projects/mireap) through mapping the precursor to goat genome sequences. Novel miRNAs presumed by exploring the secondary structure, the Dicer cleavage site and the minimum free energy of the unannotated small RNA reads. 35 potential novel miRNAs were detected in total, of which 8 potential novel miRNAs were co-expressed, 9 and 18 potential novel miRNAs were specifically expressed in the multiple and uniparous libraries, respectively (Additional file 6 and Additional file 7). The length of the novel miRNA sequences also ranged from 20 to 24 nt, and these novel miRNAs were not analyzed further, as their expression quantity were very low.

### Target gene prediction for miRNAs

miRNAs modulate gene expression by inhibiting mRNA translation or regulating mRNA degradation at the post-transcriptional level based on pairing between the 5’ end of the miRNA (i.e., 2-8 nt, the“seed”region) and the 3’ untranslated regions (3’ UTR) of target mRNAs [1, 38–40]. Mireap software was used to predict target genes of the miRNA by searching the goat reference gene database (http://www.ncbi.nlm.nih.gov/genome/10731). In the multiple library, 3,091,082 target sites in 22,171 target genes were predicted for 1,317 conserved miRNAs, and 41,781 target sites in 17,714 target genes were predicted for 17 novel miRNAs. In the uniparous library, 3,427,044 target sites in 22,171 target genes were predicted for 1,441 conserved, and 67,990 target sites into 19,812 target genes were predicted for 26 novel miRNAs.

### Gene Ontology (GO) enrichment and KEGG pathway analysis of target genes

GO is an international standarized classification system for gene function, which supplies a set of controlled vocabulary to comprehensively describe the property of genes and gene products. There are 3 ontologies in GO: cellular component, molecular function and biological process. The basic unit of GO is GO-term, each of which belongs to one type of ontology. In this study, GO enrichment analysis was used for predicting candidate target genes of all detected miRNAs. In Additional file 8, GO enrichment for gene background based on the cellular component showed that 13,973 genes were mapped to GO terms in the database (http://www.geneontology.org/). For all miRNAs target genes of multiple and uniparous goats in the ovaries during follicular phase, there were 11,577 and 12,767 target genes mapped to the GO terms of cellular component. Compared to the reference gene background, 2 and 4 GO terms were significantly (P-value < 0.05) enriched for multiple and uniparous libraries respectively based on the cellular component. Analysis of molecular function showed that 13,013 genes were assigned different functions based on gene background, while 10,806 and 11,902 target genes were involved for multiple and uniparous libraries. Compared to the reference gene background, 11 and 16 GO terms were significantly (P-value < 0.05) enriched for multiple and uniparous libraries respectively based on molecular function. For the reference gene background, 13,113 genes were related to biological processes. However, 10,877 and 11,969 target genes were related to the biological processes of GO terms for multiple and uniparous libraries. Compared to the reference gene background, 1 and 4 GO terms were significantly (P-value < 0.05) enriched for multiple and uniparous libraries respectively based on biological processes.

KEGG pathway annotation showed that 16,155 background genes were annotated for 309 biological functions. However, 13,472 and 14,860 target genes were annotated to the relevant biological functions for multiple and uniparous goats in the ovaries during follicular phase. The only over-represented miRNA targets belonged to the olfactory transduction pathways in uniparous library, while there 10 pathways were significantly (P-value < 0.05) enriched in multiple library (Additional file 9).

### Quantitative RT-PCR validation

**Figure 4:**
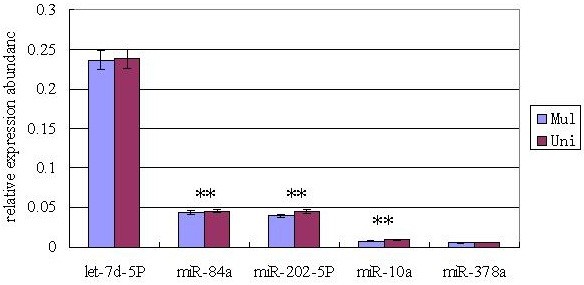
**RT-PCR validation of miRNAs identified in goat ovaries using Solexa sequencing technology.** Note: **indicate the significant (P < 0.01) difference in expression level between multiple and uniparous goats by GLM of SAS software.

The expression levels of 5 (miR-378a, miR-10a, miR-202-5p, miR-84a, and let-7d-5p, and 3 of them were differentially expressed) randomly selected miRNAs were verified in the ovaries of multiple and uniparous goats during follicular phase using RT-PCR. The relative expression levels of 5 selected miRNAs were consistent with the Solexa sequencing results since they had a similar trend of expression in two libraries (Figure 4).

## Discussion

Economic efficiency of a flock of goats is dependent on its total productivity, and the productivity is more dependent on fertility and prolificacy of the female goats than any other components [41–43]. However, hircine fecundity is relatively low [13], and the trait is difficult to improvement by conventional breeding methods for its low heritability which ranged 0.09 to 0.14 based on the study of Zhang et al. [43]. Therefore, researchers pin their hope on molecular assisted breeding technology, and the research of miRNA in reproduction thrived [2, 9, 13, 27, 35]. In this study, we sequenced the small RNAs in the ovarian tissues of multiple and uniparous Anhui White goats during follicular phase by Illumina Solexa technology, then analyzed the differentially expressed miRNAs, predicted novel miRNAs, and made GO enrichment and KEGG pathway analysis of target genes in two miRNA libraries.

Compare to researches carried by predecessors [36], more conserved miRNAs (1317 and 1441 in the multiple and uniparous libraries) were identified in this study. There are two principal reasons contributing to these results. The first reason is that the detected clean reads was aligned to the latest database of miRBase 20.0 released in June 2013, while 3355 new hairpin sequences and 5393 new mature microRNAs from around 40 new publications were added to the database based on miRBase 19.0, increasing the totals to 24521 hairpin sequences and 30424 mature sequences in all (http://www.mirbase.org/blog/). And the miR/miR* nomenclature is finally replaced by the -5p/-3p nomenclature after miRBase 18.0, the result leading to miRNA* which was not reckoned in real conversed miRNA once, now named -5p or -3p; ultimately duplex-like miRNA:miRNA* what was regarded to be one miRNA turned out to be two. The second reason is that the two libraries were compared with all animals in the miRBase 20.0 for there is no miRNAs information of goat in it, and the reference data was species widely.

In ovaries between multiple and uniparous goats of follicular phase, 35 novel miRNAs were predicted in total, which is distinctly more than the amount predicted in our previous study (ovaries from pregnant and non-pregnant goats) implemented by our team workers, Zhang et al. [13]. For this result, a central factor is should be attributed to the new reference of goat genome which published in December 2012 when the study of Zhang et al. had been completed, and they simply identified novel miRNAs by means of alignment with goat expressed sequence tags (ESTs). It worth to point out is that 1 (from multiple libraries) of the 35 novel miRNAs sequence is consistent with the sequence (from non-pregnant libraries) predicted in our previous study. This result may hint that the sequence should be a greater likelihood of a potential new miRNA.

In sheep, selection for superior lambing rate has been showed to alter ovulation rate primarily [44]. And it has been suggested that increased ovulation rates could be due to a wider window of time for follicle recruitment or an increase in the numbers of follicles recruited in ovaries [45]. To find out the different ovarian activity and follicle recruitment in multiple and uniparous goats of follicular phase, differentially expressed miRNA were identified in the two constructed libraries. The 20 most highly expressed miRNAs in the multiple library were mainly consistent with that in the uniparous library, and there were no significant difference in expression between two libraries. This result was ascribed to the same physiological phase (follicular phase) in ovaries of two experimental groups. MiR-21 was verified in regulation of apoptosis in vivo, and related with ovulation rate [46]. However, the expression of miR-21 also have no significant difference between multiple and uniparous groups in this study. Then, we turned our attention to the specific expressed miRNAs. The highest specific expressed miRNA in multiple library was miR-29c, and the one in uniparous library was miR-6406. As aligning the clean reads to the miRNA precursor/mature miRNAs of all animals in the miRBase 20.0 database, and obtained miRNA with no specifid species. Carefully analyzed miR-29c and miR-6406 in the present study, we found that the sequence of miR-29c was consistent with ola-mir-29c (from Oryzias latipes), the sequence of miR-6406 endured some mismatch with mmu-mir-6406 (from Mus musculus). miR-29c was in the same family with MiR-29a, which was significantly down-regulated after 12 h FSH treatment, while its expression increased after 48 h FSH treatment [47]. Therefore, it was supposed that miR-29c was also related with FSH secretion which may influence on follicles recruiting in the present study, and it need further experimentation certainly. As for miR-6406, there is no research reported on ovary so far and miRBase recorded the mmu-mir-6406 by reference the article of David [48]. In consideration of specific and higher level expression of miR-6406 in uniparous group, some further experimentation also worth to be done.

GO annotation and KEGG Pathway analyses are able to obtain a better understanding from the cellular components, molecular functions and biological processes of target genes [10]. Start with GO analysis, miRNA targets were significantly enriched to different terms in two libraries, and the significantly enriched terms in uniparous library contained the terms in multiple library. As for KEGG pathway analysis, it is worth to note that Focal adhesion, ABC transporters, Carbohydrate digestion and absorption, Peroxisome, Starch and sucrose metabolism, and Progesterone-mediated oocyte maturation were involved in the significantly enriched pathway in the ovaries during follicular phase of multiple goats. These significantly enriched pathways may imply that the organism was coping to the criteria of follicular phase in multiple goats. GO annotation and KEGG Pathway analyses can provide a reference to us for the later research.

RT-PCR was carried out to analyze the expression of 5 randomly selected miRNAs in multiple and uniparous hircine ovaries during follicular phase, and the results were consistent with the Solexa sequencing data. However, the expression levels of every miRNA need to be validated by RT-PCR in theory. Hence, the identified miRNAs in the present study can only be regard as a hircine ovary-specific miRNA reference dataset. Compared with previous study implemented by Zhang et al. in our team [13], which the ovaries tissues were from different physiological phase of pregnant and non-pregnant phase in the same goats, while the ovaries tissues in this study were from different goat of multiple and uniparous goats in the same follicular phase. Therefore, the results of our previous study were caused by the different physiological phase, while the results of our present study were caused by the different genetic background. Nevertheless, the potential of kidding rate is affected by many components, including ovulation rate, fertilization rate and embryo survival, any or all of which may be under genetic control [49], and these need to be researched step by step.

## Conclusions

In summary, 1008 miRNAs were co-expressed, 309 and 433 miRNAs were specifically expressed in the ovaries of multiple and uniparous goats during follicular phase. The highest specific expressed miRNA in multiple library was miR-29c, and the highest specific expressed miRNA in uniparous library was miR-6406. 35 novel miRNAs were predicted in total. GO annotation and KEGG Pathway analyses were implemented on target genes of all miRNA in two libraries, Progesterone-mediated oocyte maturation and other pathways were pointed out for significantly enriched. The result may help to further understand the role of miRNAs in kidding rate regulation and also help to identify miRNAs which could be potentially used to increase hircine ovulation rate and kidding rate in the future.

## Methods

### Animals and sample preparation

The experimental goats of this study, Anhui White goats (a Chinese indigenous breed) were obtained from the College of Animal Science and Technology, Anhui Agricultural University, Hefei, China. The ovaries of Anhui White goats were collected and froze in liquid nitrogen instantly then stored at -80°C for generating small RNA libraries. 6 target goats, 3 were 3-year old multiple goats whose litter size was more than one (Mul) and the other were 3-year old uniparous goats whose litter size was only one (Uni), accepted the teasing behavior were chosen as our experimental samples for their ovaries. Mul: the three goats had three litters which kidding ≥ 2. Uni: the three goats had three litters which kidding = 1. All the experimental procedures with Anhui White goats used in the present study had been given prior approval by the ethics committee of Anhui Agricultural University, Anhui, China, under permit No. AHAU20101025.

### Small RNA library construction and sequencing

Two groups of total RNA were used for library preparation and sequencing by pooling equal quantity (10 μg) of total RNA isolated from six individual multiple and uniparous goats ovaries. Small RNA fragments of 18-30 nt in length were isolated and purified from total RNA using 15% denaturing polyacrylamide gel electrophoresis (PAGE). Subsequently, a 3’ RNA adaptor and 5’ RNA adaptor were ligated to the RNA pool using T4 RNA ligase, then the samples were used as templates for cDNA synthesis. The cDNAs were amplified using the appropriate number of PCR cycles to produce sequencing libraries, which were subsequently subjected to the proprietary Solexa sequencing-by-synthesis method using the Illumina Genome Analyzer (SanDiego, CA, USA) at the Beijing Genomics Institute (BGI, Shenzhen, China).

### Sequence analysis

According to the requirement of this experiment and the principle of bioinformatics analysis, some contaminant reads should be removed from the raw reads, such as low quality reads and reads with 5’ primer contaminants, reads without 3’ primer, reads without the insert tag, reads with poly (A), and reads shorter than 18 nt. Then the final clean reads for summarizing the length distribution and counts were got, all valid sequences were remained for further analysis. The clean reads were compared with the ncRNAs (rRNAs, tRNAs, snRNAs, and snoRNA) deposited in the NCBI GenBank database and the Rfam10.1 database using BLAST to annotate the sRNA sequences. The clean reads were also mapped to the goat genome (http://goat.kiz.ac.cn/GGD/download.htm) by SOAP v1.11 to analysis their expression and distribution in the goat genome. The clean reads was aligned to the miRNA precursor/mature miRNA of all animals in miRBase 20.0 (http://www.mirbase.org/), show the sequence and count of miRNA families (no specific species) which can be found in the samples. According to the characteristic hairpin structure of miRNA precursor can be used to predict novel miRNA. The unannotated sequences were used to predict potential novel miRNA candidates by Mireap (http://sourceforge.net/projects/mireap/) mapped to the goat genome.

### Differential expression analysis of two libraries

Comparing the known miRNA expression between two libraries (Mul and Uni) to find out the differentially expressed miRNAs, Log2-ratio figure and Scatter Plot were plotted. Procedures were shown as below: (1)Normalize the expression of miRNA in two libraries to get the expression of transcript per million (TPM). Normalization formula: Normalized expression = Actual miRNA count/Total count of clean reads*1000000; (2) Calculate fold-change and P-value from the normalized expression. Then generate the Log2-ratio figure and Scatter Plot. Fold-change formula:

P-value formula:

The x and y represent normalized expression level, and the N1 and N2 represent total count of clean reads of a given miRNA in small RNA library of ovaries of multiple and uniparous goats, respectively [35].

When the normalized expression of a certain miRNA was zero in one of the two libraries, its expression value was revised to 0.01. If the normalized expression of a certain miRNA in two libraries was all lower than 1, further differential expression analysis was conducted without this miRNA for the reason of its low expression.

### GO enrichment and KEGG pathway analyses

GO enrichment analysis of present study was the best on predicted target gene candidates of all detected miRNAs compared to the reference gene background, as well as the genes corresponding to certain biological function. The result could reveal the functions significantly related with predicted target gene candidates of all detected miRNAs. This method firstly mapped all target gene candidates to GO terms in the database (http://www.geneontology.org/), calculated gene numbers for each term, then used hyper geometric test to find significantly enriched GO terms in target gene candidates compared to the reference gene background. The calculating formula is:

In the formula above, N is the number of all genes with GO annotation; n is the number of target gene candidates in N; M is the number of all genes that were annotated to a certain GO term; m is the number of target gene candidates in M. The Bonferroni Correction for the p-value was used to obtain a corrected p-value. GO terms with corrected p-value ≤ 0.05 are defined as significantly enriched in target gene candidates. This analysis could recognize the main biological functions for target gene candidates.

The same as Gene Ontology, KEGG pathway analysis is also based on the target gene candidates. In organisms, genes usually interact with each other to play different roles in certain biological function. KEGG pathway analysis could facilitate the understanding of biological functions of genes. KEGG is a major public pathway-related database [50]. KEGG pathway analysis identifies significantly enriched metabolic pathways or signal transduction pathways in target gene candidates comparing with the whole reference gene background. The calculating formula is the same as that in GO analysis. Here N is the number of all genes with KEGG annotation, n is the number of target gene candidates in N, M is the number of all genes annotated to a certain pathway, and m is the number of target gene candidates in M. Genes with FDR ≤ 0.05 are considered as significantly enriched in target gene candidates. The KEGG analysis could reveal the main pathways which the target gene candidates are involved in.

### MiRNA validation via RT-PCR

For validating the Solexa sequencing data, RT-PCR assay was carried out by five randomly selected miRNAs. One microgram of total RNA from each sample were reverse-transcript into cDNA using the miScript Reverse Transcription Kit (Qiagen, Dusseldorf, Germany) according to the manufacturer’s instructions. The template for RT-PCR was got, after incubation at 37°C for 1 h and deactivation at 95°C for 5 min. The reaction system of RT-PCR contained 2.0 μl cDNA, 32.5 μl SYBRGreen Mix (Thermo, Shanghai, China), 0.5 μl of each primer and 14.5 μl H_2_O. RT-PCR was performed using standard protocols on the Roche LightCycler 480 II Real-Time PCR Detection System (Roche; LC480 II, Basel, Switzerland). The reaction was incubated at 95°C for 10 min, followed by 40 cycles of 95°C 15 s and 60°C 45 s. All reactions were performed in triplicate. The threshold cycle (CT) was collected from each reaction, and the relative expression level of each miRNA to 5S snRNA was evaluated using the equation 2^-(CTmiRNA-CT5SRNA)^. GLM was used to examine the significance of the expression in two samples by SAS 8.0 software. The miRNA specific primers were presented in Additional file 10.

## Electronic supplementary material

Additional file 1: **The flowing results of data filtration and the distribution of sequenced small RNAs.** (XLS 10 KB)

Additional file 2: **Frequency distribution of sequence lengths of the unann reads.** (DOC 116 KB)

Additional file 3: **Conserved miRNAs in the ovaries of multiple and uniparous goats during follicular phase.** (XLS 342 KB)

Additional file 4: **Differential expression of conserved miRNAs in the ovaries of multiple and uniparous goats during follicular phase.** (1). -std. represents normalized expression level of miRNA in a sample. Normalized expression = Actual miRNA count/Total count of clean reads*1,000,000. (2). Sig-label: ** represents fold change (log2) > 1 or fold change(log2) < -1, and p-value < 0.01; * represents fold change(log2) > 1 or fold change(log2) < -1, and 0.01 ≤ p < 0.05; None represents others. Fold change = log2(Pregnant std./Non-pregnant std.) (3). miRNAs in red font used for the RT-PCR analysis. (XLS 176 KB)

Additional file 5: **Clustering of miRNAs differentially expressed during follicular phase in ovaries.** (PNG 2 MB)

Additional file 6: **Information of the potential novel miRNAs on goat.** (XLS 74 KB)

Additional file 7: **The stem loop structures of precursors of predicted miRNA candidates.** (DOC 280 KB)

Additional file 8: **GO enrichment analysis for the target genes of conserved miRNAs.** (XLS 5 MB)

Additional file 9: **KEGG pathways for the target genes of all detected miRNAs.** (XLS 124 KB)

Additional file 10: **Primer sequences for RT-PCR experiments.** (XLS 9 KB)

## Abbreviations

miRNA: microRNA
HF: Hair follicle
sRNA: Small RNA
UTR: Untranslated regions
GO: Gene ontology
KEEG: Kyoto encyclopedia of genes and genomes
RT-PCR: Reverse transcription PCR
BGI: Beijing genomics institute
MFE: Minimum free energy
TPM: Transcript per million
GLM: General linear model
SAS: Statistical analysis system.
