# Supplementary figures and images for: Identification and characterization of microRNAs in the ovaries of multiple and uniparous goats (Capra hircus) during follicular phase
Source: BMC Genomics. 2014 May 6;15(1):339. doi: 10.1186/1471-2164-15-339 (PMC4035069; doi:10.1186/1471-2164-15-339)

**Additional file 2: Frequency distribution of sequence lengths of the unann reads**


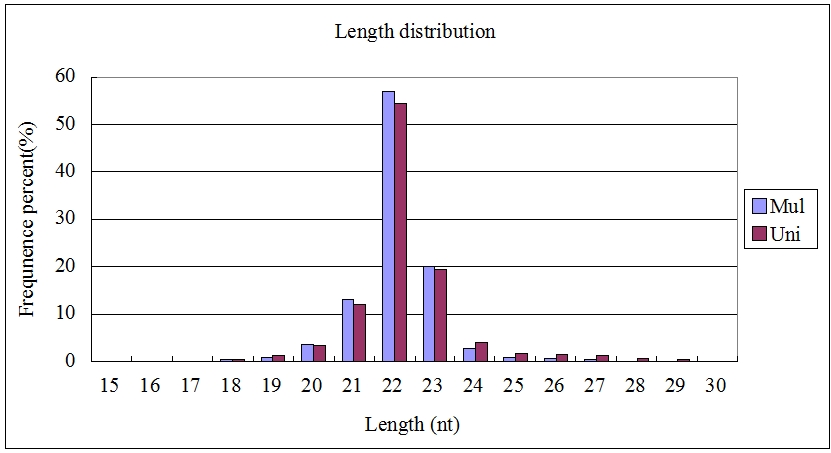

Supplement: Supplementary file 2 — Additional file 2: Frequency distribution of sequence lengths of the unann reads. (DOC 116 KB) [file 12864_2014_6036_MOESM2_ESM.doc]
