# Supplementary material for: Identification and characterization of microRNAs in the ovaries of multiple and uniparous goats (Capra hircus) during follicular phase
Source: BMC Genomics. 2014 May 6;15(1):339. doi: 10.1186/1471-2164-15-339 (PMC4035069; doi:10.1186/1471-2164-15-339)
Supplement: Supplementary file 7 — Additional file 7: The stem loop structures of precursors of predicted miRNA candidates. (DOC 280 KB) [file 12864_2014_6036_MOESM7_ESM.doc]

**Additional file7: The stem loop structures of precursors of predicted miRNA candidates**


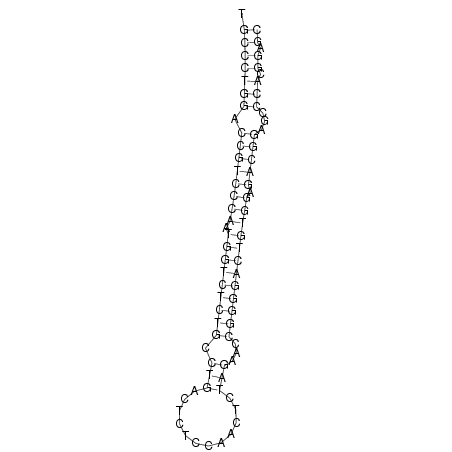


Mul-m0001


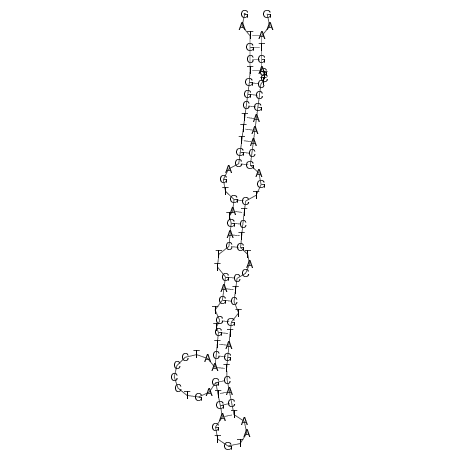


Mul-m0002


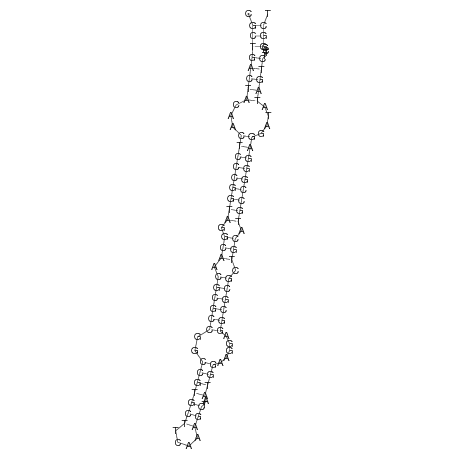


Mul-m0003


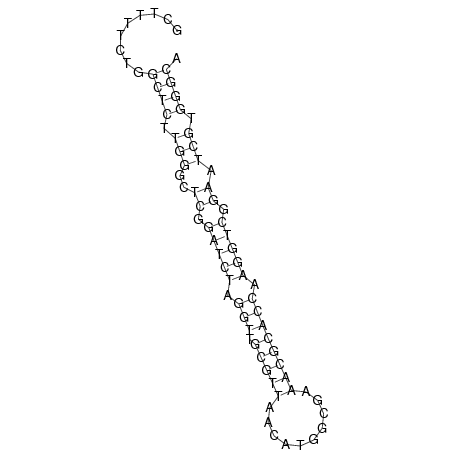


Mul-m0004


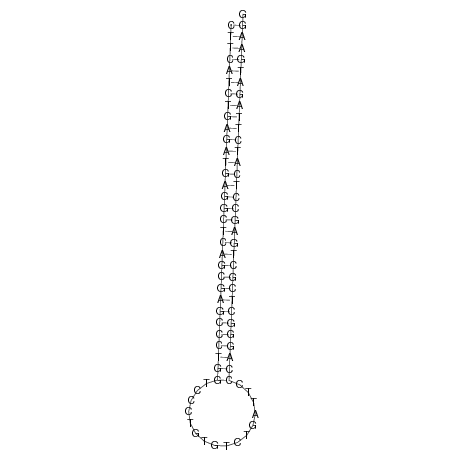


Mul-m0005


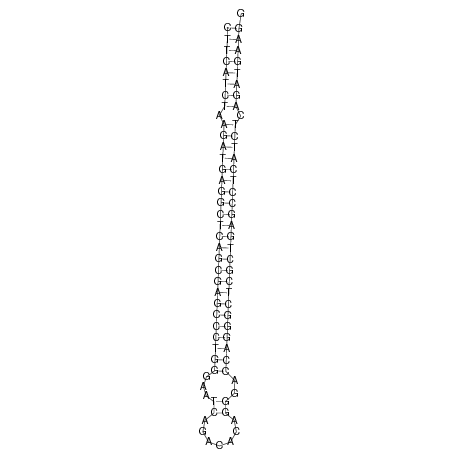


Mul-m0006


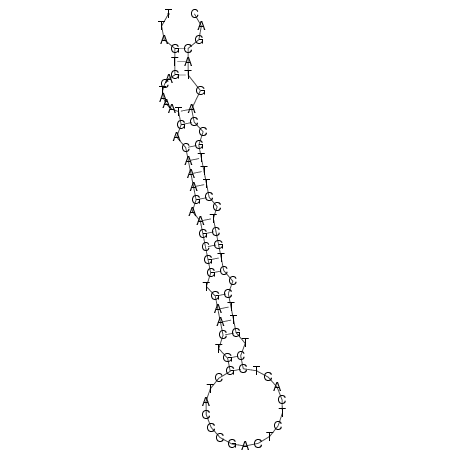


Mul-m0007


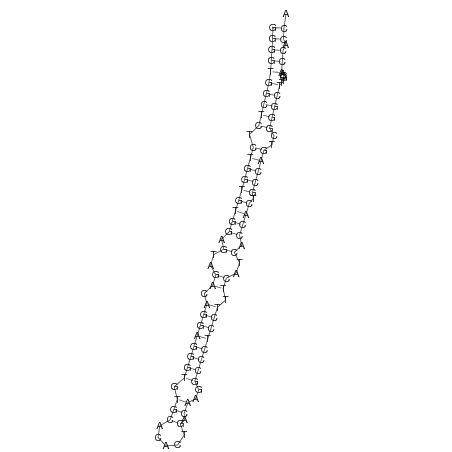


Mul-m0008


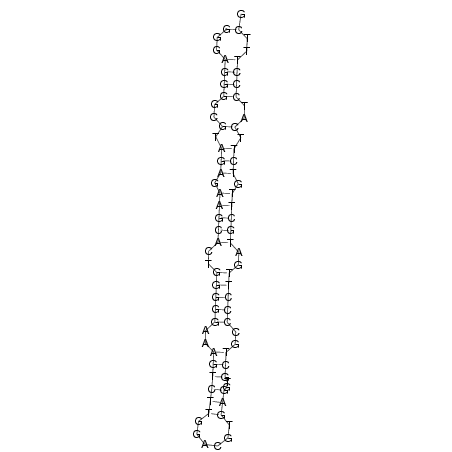


Mul-m0009


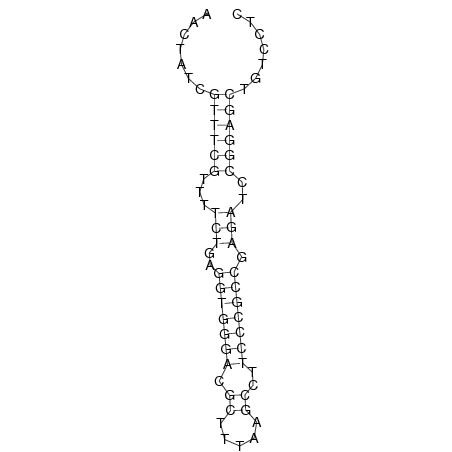


Mul-m0010


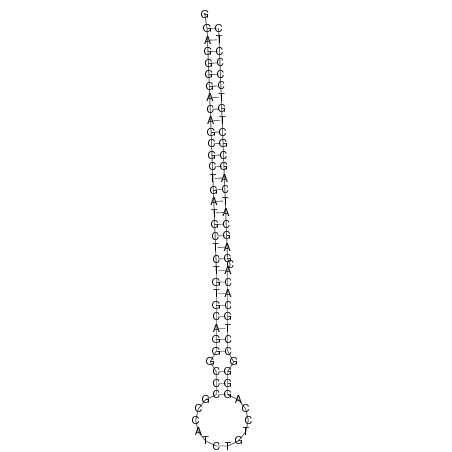


Mul-m0011


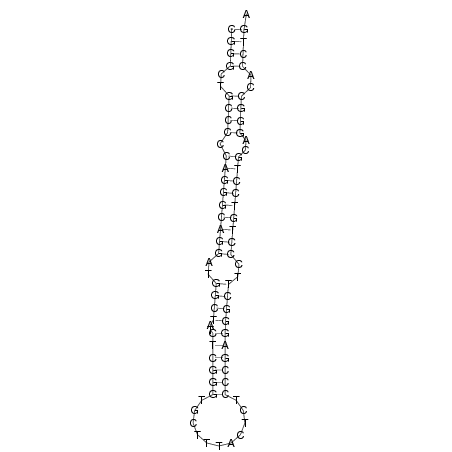


Mul-m0012


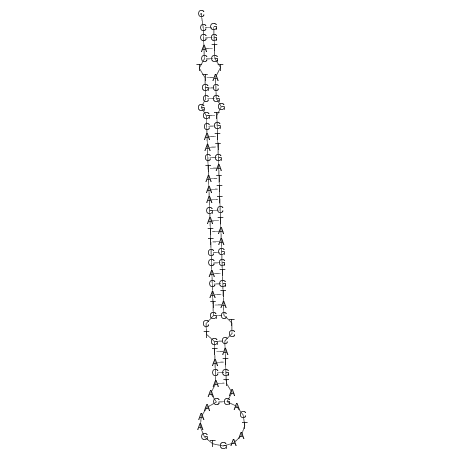


Mul-m0013


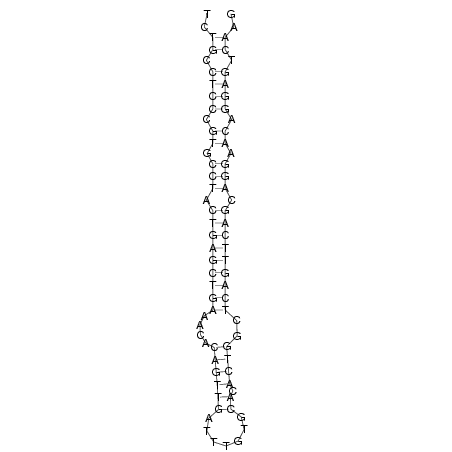


Mul-m0014


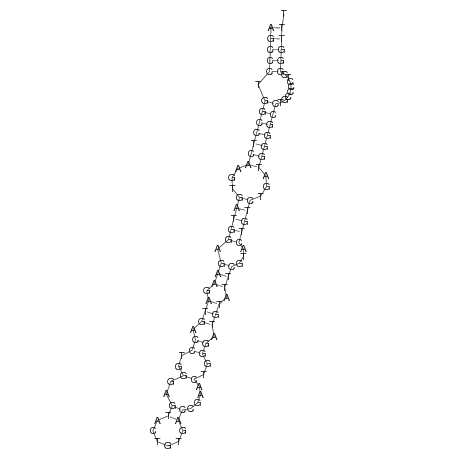


Mul-m0015


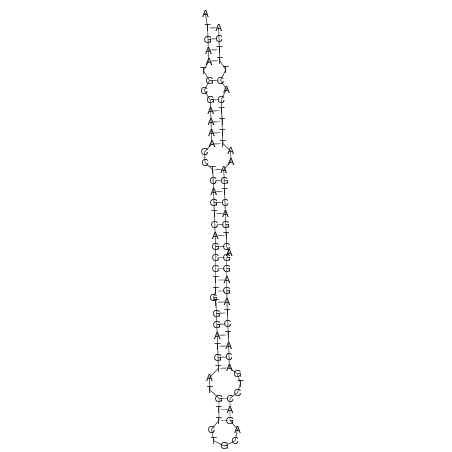


Mul-m0016


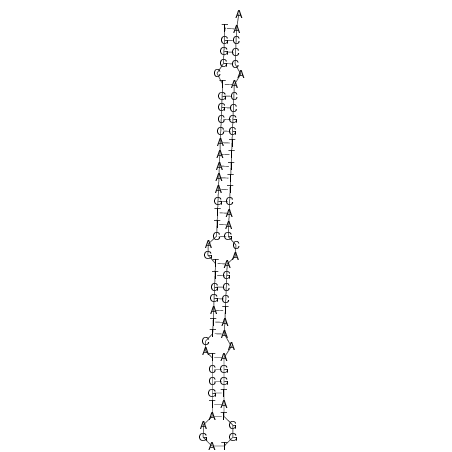


Mul-m0017


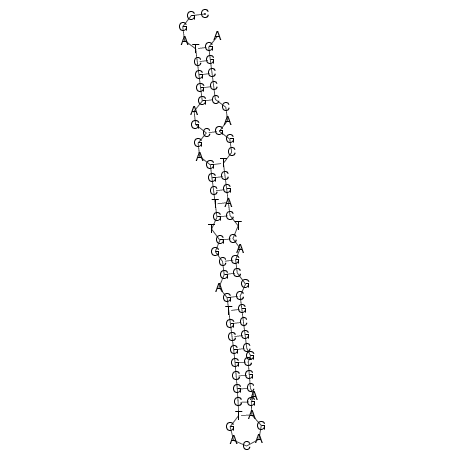


Uni-m0001


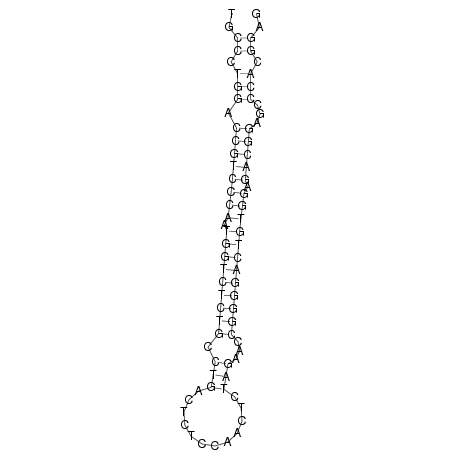


Uni-m0002


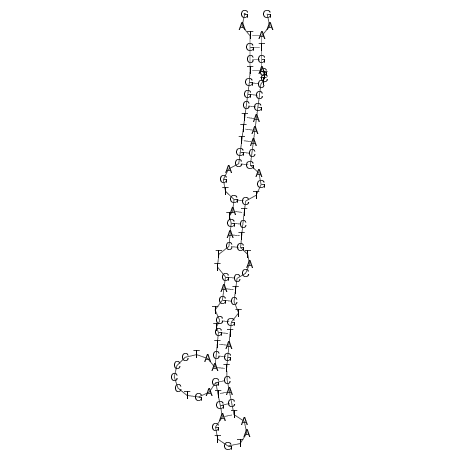


Uni-m0003


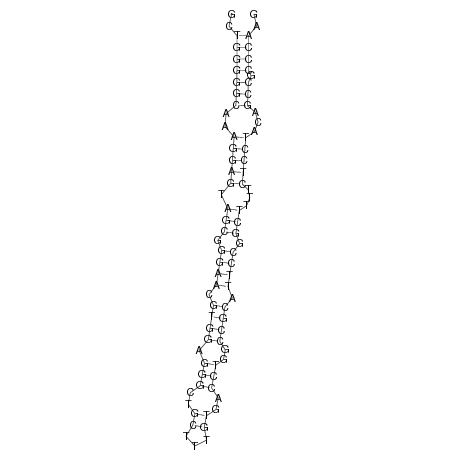


Uni-m0004


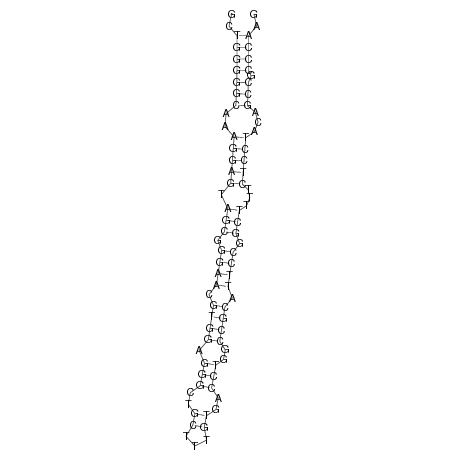


Uni-m0005


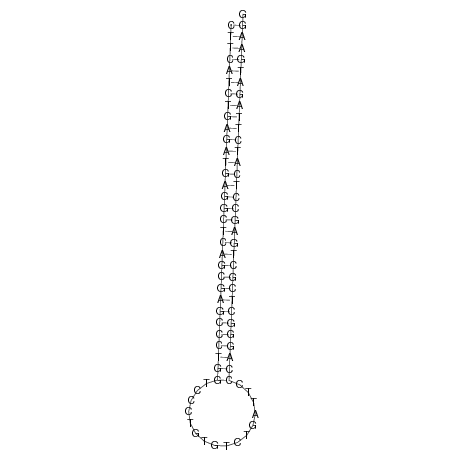


Uni-m0006


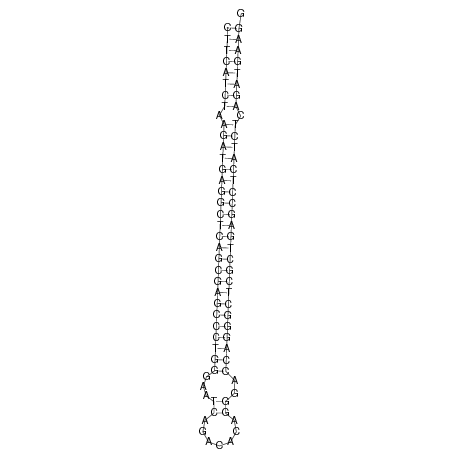


Uni-m0007


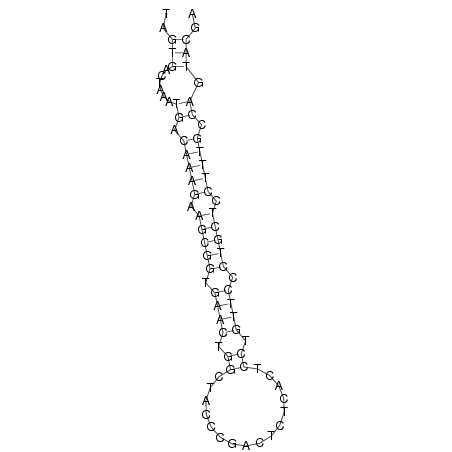


Uni-m0008


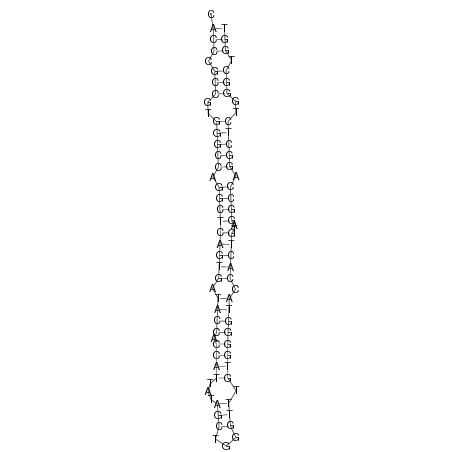


Uni-m0009


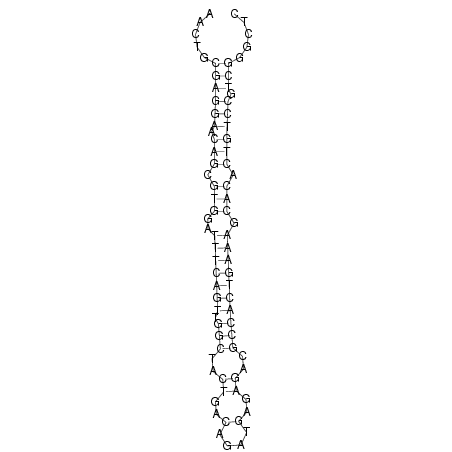


Uni-m0010


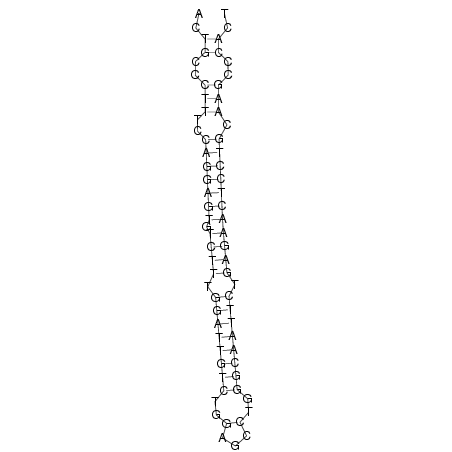


Uni-m0011


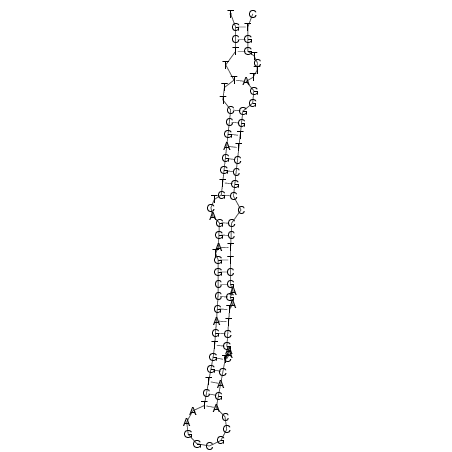


Uni-m0012


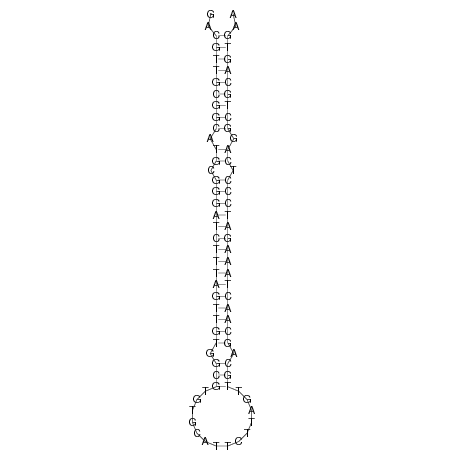


Uni-m0013


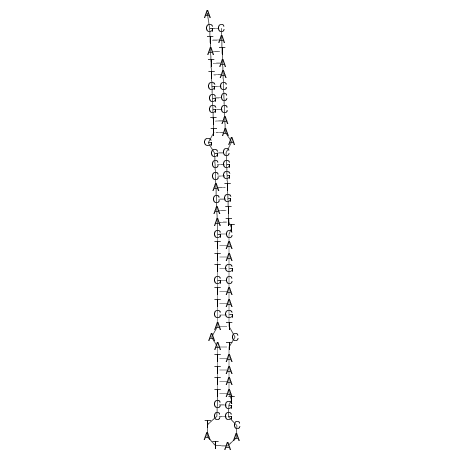


Uni-m0014


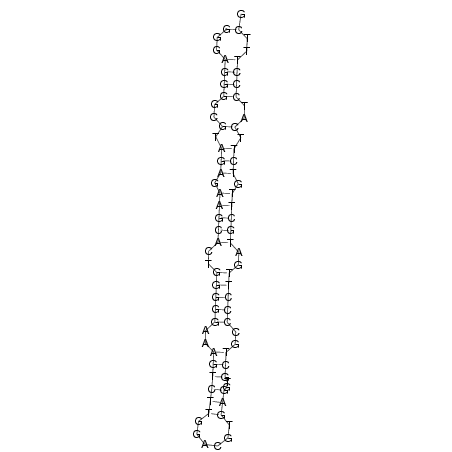


Uni-m0015


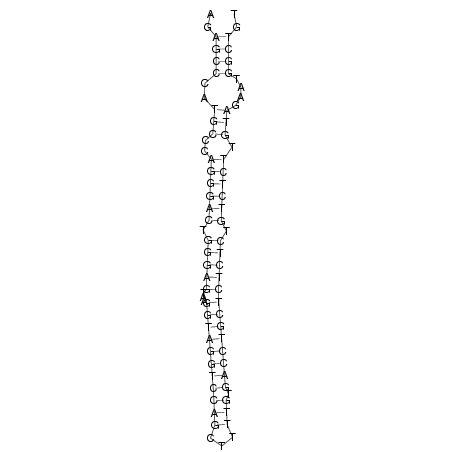


Uni-m0016


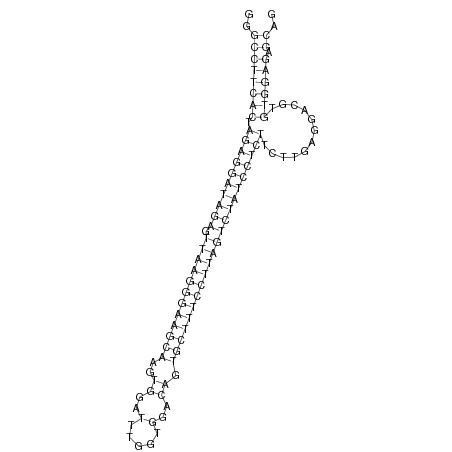


Uni-m0017


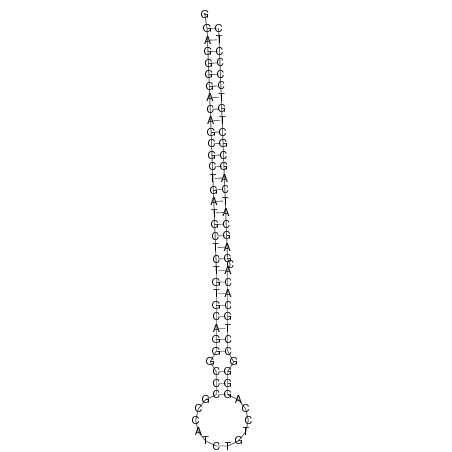


Uni-m0018


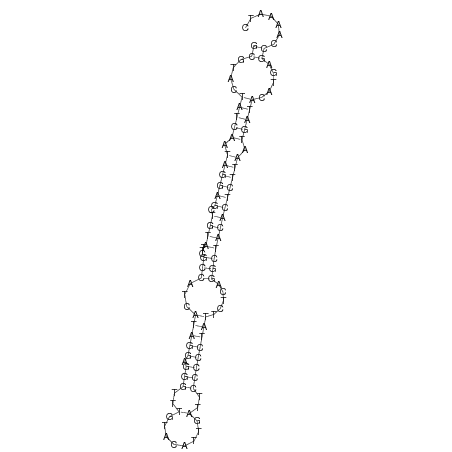


Uni-m0019


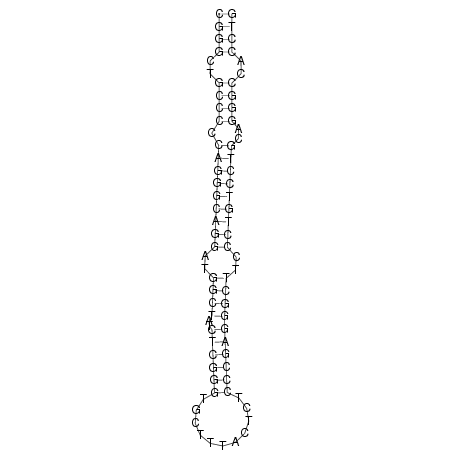


Uni-m0020


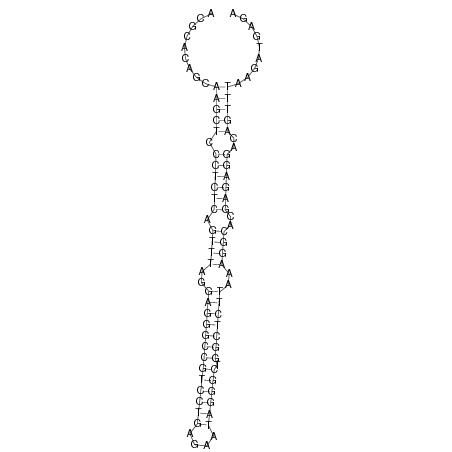


Uni-m0021


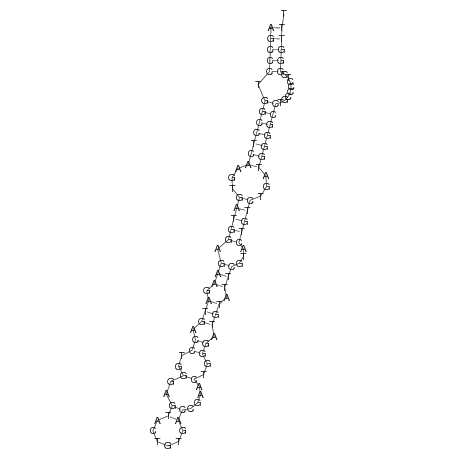


Uni-m0022


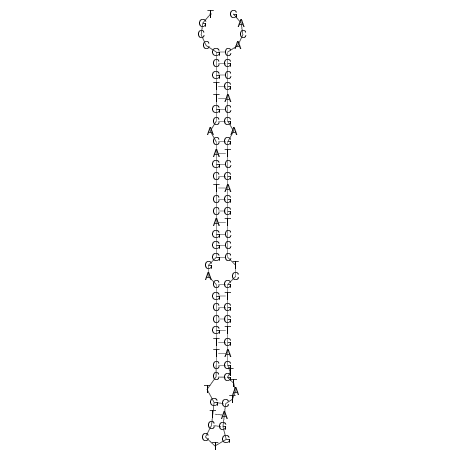


Uni-m0023


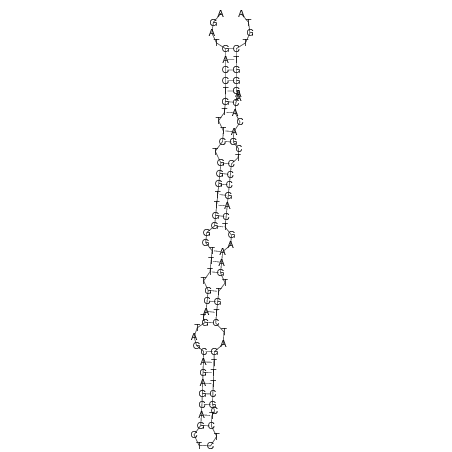


Uni-m0024


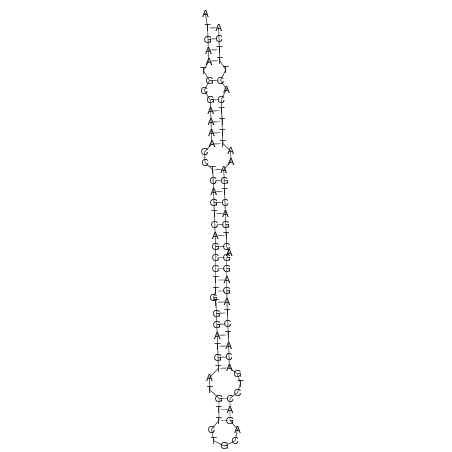


Uni-m0025


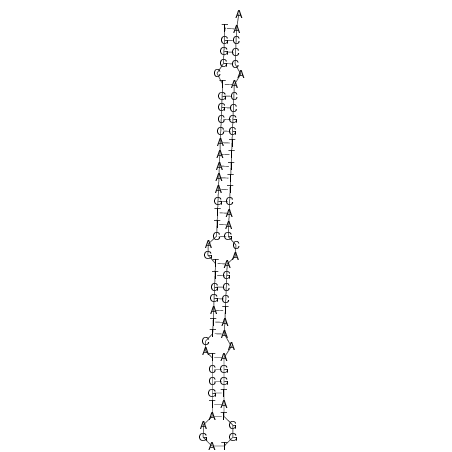


Uni-m0026
